# *Helicobacter pylori* as an Initiating Factor of Complications in Patients With Cirrhosis: A Single-Center Observational Study

**DOI:** 10.3389/fmed.2020.00096

**Published:** 2020-03-24

**Authors:** Ahmed Abdel-Razik, Nasser Mousa, Rania Elhelaly, Rasha Elzehery, Ahmad S. Hasan, Mostafa Abdelsalam, Ahmed Salah Seif, Ahmed M. Tawfik, Niveen El-Wakeel, Waleed Eldars

**Affiliations:** ^1^Tropical Medicine Department, Faculty of Medicine, Mansoura University, Mansoura, Egypt; ^2^Clinical Pathology Department, Faculty of Medicine, Mansoura University, Mansoura, Egypt; ^3^Nephrology and Dialysis Unit, Internal Medicine Department, Faculty of Medicine, Mansoura University, Mansoura, Egypt; ^4^Scientific Fellow of Tropical Medicine, Hepatology and Gastroenterology Department, Shebin Elkom Teaching Hospital, Menoufia, Egypt; ^5^Diagnostic & Interventional Radiology Department, Faculty of Medicine, Mansoura University, Mansoura, Egypt; ^6^Medical Microbiology and Immunology Department, Faculty of Medicine, Mansoura University, Mansoura, Egypt; ^7^Medical Microbiology and Immunology Department, Faculty of Medicine, Delta University for Science and Technology, Talkha, Egypt

**Keywords:** *Helicobacter pylori*, liver cirrhosis, C-reactive protein, hepatocellular carcinoma, portal vein thrombosis, spontaneous bacterial peritonitis, hepatic encephalopathy

## Abstract

**Background and Aim:** The relationship between liver cirrhosis and *Helicobacter pylori* (*H. pylori*) is a debatable matter. The aim of this study is to evaluate the possible association between *H. pylori* infection and liver cirrhosis.

**Methods:** A single-center prospective cohort pilot study of 558 patients with cirrhosis was followed up for 1 year. Serum C-reactive protein (CRP), tumor necrosis factor-α (TNF-α), interleukin-6 (IL-6), nitric oxide (NO), vascular endothelial growth factor (VEGF) levels and Fecal *H. pylori* antigen were evaluated by enzyme-linked immunosorbent assay (ELISA). All patients with positive *H. pylori* were treated and then followed up for 3 months. Participants with eradicated *H. pylori* were followed up for one further year.

**Results:** *H. pylori*-positive patients (48.4%) were associated with increased levels of serum CRP, TNF-α, IL-6, NO, and VEGF, as well as increased incidence of varices, portal hypertensive gastropathy, gastric antral vascular ectasia, hepatocellular carcinoma (HCC), spontaneous bacterial peritonitis, hepatic encephalopathy, portal vein thrombosis (PVT), and hepatorenal syndrome (all *P* < 0.05). Multivariate analysis models revealed that the presence of *H. pylori* was an independent risk variable for the development of portal vein thrombosis and hepatocellular carcinoma (*P* = 0.043, *P* = 0.037) respectively. After treatment of *H. pylori* infection, there was a significant reduction in all measured biochemical parameters and reported cirrhotic complications (all *P* < 0.05).

**Conclusion:** Incidence of PVT and HCC development increased with *H. pylori* infection through increased inflammatory markers and vascular mediators. Moreover, its eradication may reduce the incidence of these complications.

## Introduction

*Helicobacter pylori* (*H. pylori*) is a pathogenic bacteria that affects the human gastric tissue, and it is the most common etiology of peptic ulcer and gastritis (1). However, *H. pylori* is implicated in extra-gastric disorders, as idiopathic thrombocytopenic purpura, vitamin B12 deficiency, iron deficiency, and can also be related to many other diseases, for example, neurodegenerative syndromes, ischemic heart disease, and diabetes (2). Furthermore, it has been hypothesized that *H. pylori* could be a risk factor for many liver disorders, for example, nonalcoholic fatty liver disease, isolated hypertransaminasemia, and portosystemic encephalopathy (3–6).

As mentioned in the literature review, the association between liver diseases and *H. pylori* has been discussed and still remains a matter of debate (7). While the presence of *Helicobacter* species or *H. pylori* has been reported in hepatic tissue samples from patients with different hepatic disorders (8–14), nevertheless, a direct association of *H. pylori* in the development of cirrhotic complications in patients with liver disease has been postulated with a less strong evidence (3). To date, the problem has received scant attention in the research literature. The purpose of this study, therefore, is to evaluate the possible association between *H. pylori* infection and liver cirrhosis.

## Patients and Methods

This prospective single-center cohort pilot study was carried out at the Tropical Medicine Department (Mansoura University-Egypt), between April 2015 and May 2019. We prospectively enrolled 803 consecutive patients with liver cirrhosis who were referred to our center. Only 558 patients who met the inclusion criteria were included in this study. Patients' clinical, radiological, demographic, hematological, and biochemical findings were evaluated at baseline and throughout the follow-up periods.

The inclusion criteria were (1) patients with liver cirrhosis, (2) aged ≥18 years, and (3) had undergone all investigations to confirm *H. pylori* infection.

The exclusion criteria were (1) patients with history of gastrectomy, (2) had cancer, (3) had alcoholic liver disease, (4) had missing data, (5) pregnancy and lactation, (6) patients with kidney diseases and hematologic disorders; (7) pancreatitis, (8) peritoneal carcinomatosis, (9) abdominal tuberculosis, (10) uncontrolled thyroid disorders, (11) cerebrovascular accident causes, (12) bone marrow suppression, (13) had inherited coagulation abnormalities, (14) collagen vascular diseases, (15) patients with metabolic or cholestatic hepatobiliary disorders, (16) heart failure, (17) patients with portal vein thrombosis (PVT), spontaneous bacterial peritonitis (SBP), hepatocellular carcinoma (HCC), hepatorenal syndrome (HRS), and hepatic encephalopathy (HE) at baseline of the study, (18) usage of hepatotoxic, antiplatelet or anticoagulant treatment, oral contraceptive drugs, and NSAIDs, (19) who had a history of recent upper gastrointestinal (GI) bleeding (in last 6 weeks) were excluded from the study. (20) use of proton-pump inhibitors (PPIs) for at least 4 weeks before enrollment, and (21) recent usage of antibiotics and/or prophylaxis with norfloxacin or rifaximin for SBP within the preceding 3 months prior to the onset of the study were also excluded from this study.

### The First Part of the Study: Patients Fulfilling the Inclusion Criteria Follow-Up

All patients (*n* = 558) who met the criteria were followed up for 1-year. All biochemical parameters and complications of liver cirrhosis were also recorded.

### The Second Part of the Study: *H. pylori*-Positive Patients Treatment and Follow-Up

After a 1-year follow-up, all patients with *H. pylori* infection (*n* = 270) were treated with two regimens according to the clinical status, patient's general condition, and possible adverse effects related to drugs. The first one; pantoprazole 40 mg, clarithromycin 500 mg, and amoxicillin 1000 mg, twice daily, were used for 14 days. The second one; pantoprazole 40 mg and amoxicillin 1 g twice daily, and levofloxacin 500 mg once daily, were used also for 2 weeks. Treatment with pantoprazole (dose as mentioned above) was given to all patients for 1 more month, to complete the eradication therapy (15). All patients have been notified of the side effects of the drugs used. Therapeutic compliance was checked by tablet counting and all the patients were given an emergency telephone number, though no complications were reported leading to treatment discontinuation during the study period. To evaluate the effect of *H. pylori* eradication, all parameters were assessed again in patients with successful *H. pylori* eradication (*n* = 212) after a 3-months follow-up.

Patients who had not responded to any of the two above mentioned *H. pylori* therapeutic protocols (*n* = 58, 21.5% of patients) have received another *H. pylori* regimen used in our outpatient clinics (15).

### The Third Part of the Study: Follow-Up of all Patients With Successfully Eradicated *H. pylori*

All patients (*n* = 205; as seven patients were lost during the follow-up period) who achieved eradication were followed up for one further year in comparison with *H. pylori*-negative patients (*n* = 276; as 12 patients were lost during the follow-up period). All biochemical parameters and complications of liver cirrhosis were also recorded again.

The baseline data was captured within 1 week from the time of enrollment and the after-treatment data was captured 3 months after finishing therapy. The end-of-study data was captured in the last week of the 1 year after therapy follow up period. At the end of the study, clinical examination, radiological and biochemical findings of the patients did not show any problems affecting the follow-up parameters in the subjects' health that were not recorded at the start of the study.

### Diagnosis of Liver Cirrhosis and Its Complications

Liver cirrhosis was assessed by clinical evaluation, unequivocal biochemical results, abdominal ultrasonography (US), elastography, histopathological evaluation of liver biopsy, or endoscopic results suggesting portal hypertension. The severity of liver cirrhosis was scored according to the Child-Pugh classification and MELD scoring system (16).

A color Doppler ultrasonography using a 3.5–5 MHz convex probe (SonoAce X6 Ultrasound System; Medison Electronics, Seoul, Korea) was done to all patients. Once, PVT was suspected by routine Doppler US examination, the final diagnosis was approved by portal angiography that also enabled a more precise differentiation between complete and partial obstructive thrombosis and their extension (17). Treatment and follow-up of all patients with PVT were carried out according to EASL clinical practice guidelines (18).

Diagnosis of SBP was based on positive ascitic fluid bacterial culture and/or ascitic fluid absolute polymorphonuclear leukocyte (PMN) count of at least 250 cells/mm^3^ without an intra-abdominal source of infection (19). According to EASL guidelines, patients with suspected SBP received an appropriate empirical antibiotic therapy, and then antibiotic was shifted depending on the results of culture and sensitivity as well as secondary antibiotic prophylaxis in patients with cirrhosis with a prior history of SBP was recommended (20).

In all cirrhotic individuals, US imaging and alpha-fetoprotein (AFP) were utilized every 6 months for surveillance purposes. Two imaging systems, both showing a focal lesion >2 cm in diameter with highlights of arterial hypervascularization, or a solitary radiologic study with these highlights joined with a serum AFP level of >400 ng/ml may be considered as noninvasive diagnostic tools especially for earlier stages of HCC (21). Surveillance and management of patients with HCC were carried out according to EASL guidelines (22).

HRS was diagnosed according to the International Club of Ascites-Acute Kidney Injury (ICA-AKI) criteria (23).

Overt HE is diagnosed depending on excluding other etiologies of altered mental status and by clinical findings (24). The West Haven criteria are considered the cornerstone for classifying the severity of overt HE for most patients (25). The minimal hepatic encephalopathy (MHE) is diagnosed based on specific psychometric testing (26). The management of patients with HE was recommended according to the recent EASL guidelines (24). In this study, HE may be considered as overt HE and/or MHE.

It is generally recommended that all patients with cirrhosis at the time of diagnosis undergo elective esophagogastroduodenoscopy (EGD) screening for varices and periodically thereafter if small or no varices are identified. The New Italian Endoscopic Club has categorized the severity of portal hypertensive gastropathy (PHG) depending on the presence of 4 elementary lesions: mosaic-like pattern, red point lesions, cherry red spots, and black-brown spots, that are typically localized in the gastric mucosa related to the corpus or fundus of the stomach (27). Gastric antral vascular ectasia (GAVE) is characterized by red spots or patches in either a linear or diffuse array in the gastric antrum (28). For EGD, an Olympus GIF-Q200 (Olympus Optical Co. Ltd., Tokyo, Japan) was used.

### Diagnosis and Follow-Up of *H. pylori*

In all patients, the fecal antigen test was used to establish the diagnosis of *H. pylori* infection as well as to prove its eradication. This test was done one month after antibiotic treatment completion and 2 weeks after stopping therapy with pantoprazole (29). This test was also repeated at the end of therapy to identify any reinfection. Many types of research affirmed that the monoclonal antibodies utilization may have the highest sensitivity and specificity before and after eradication treatment (30, 31), and may also increase the diagnostic value in post-treatment testing and decrease the inter-test variability problem (32).

### Data Collection

All patients completed a standardized, self-validated questionnaire from which we collected data about their employment condition, marital status, crowding index, income, alcohol consumption, medical history; especially diabetes, malignancy and hypertension, and smoking habits, as well as previous therapeutic history. BMI was calculated as weight (kg) divided by height squared (m^2^). Crowding index is the number of individuals/rooms excluding both the kitchen and bathroom, which correlated inversely with socioeconomic status (33).

A professional nutritional team started counseling to the patients with cirrhosis and malnutrition for proper protein and calorie intake. They instructed them to ingest the recommended daily protein intake of 1.5 g/kg of actual body weight. Vitamins and micronutrients were administered to treat clinically suspected or confirmed deficiencies. Patients with cirrhotic ascites following sodium restriction had 5 g of salt added daily to their diet to improve its palatability based on EASL guidelines of recommended daily sodium intake of 80 mmol/ day in these cases (34).

### Sampling

Following overnight fasting, fresh venous blood samples (7 mL) were collected from all patients [5 mL with no anticoagulants for serum testing and 1 mL on EDTA for complete blood count (CBC)]. The serum samples were then divided into aliquots and frozen at −20°C until subsequent evaluations. Three ml of venous blood were collected into plain tubes. Samples were then transported immediately to the laboratory, separated within 15 min of collection and analyzed immediately for serum ammonia.

### Methodology

Complete liver function tests and serum creatinine were done on a Dimension Xpand Plus Chemistry Analyzer using respective kits (by Siemens Technology, Princeton, New Jersey). Serum C-reactive protein (CRP) was assessed on COBAS C111 using its standard kits (Roche Diagnostics, Basel, Switzerland). Interleukin-6 (IL-6) and tumor necrosis factor-α (TNF-α) were measured by ELISA utilizing kits supplied by Dia Source (Rue du Bosquet, Louvain-la-Neuve, Belgium. Serum nitric oxide (NO) was evaluated using ELISA kits provided by MyBiosource (San Diego, CA 92195–3308, USA), with its detection range 1.56 nmol/ml−100 nmol/ml (nmol/ml = μmol/L). Serum VEGF was assessed by ELISA kits provided by Invitrogen Corporation (Flynn Road, Camarillo, CA, USA) with its detection range (0–1.5 ng/ml). Estimated glomerular filtration rate (eGFR) was assessed according to the following equation = 186 x (Creatinine/88.4)^−1.154^ × (Age)^−0.203^ × (0.742 if female) x (1.210 if black) (35). Serum ammonia was measured using a Colorimetric kit supplied by Elabscience (14780 Memorial Drive, Suite 216, Houston, Texas 77079, USA). Assessment of *H. pylori* antigens in stool was done by a sandwich ELISA technique using specific monoclonal antibodies for *H. pylori* from Immundiagnostik AG (Stubenwald-Allee 8a, 64625 Bensheim, Germany). Based on their absorbances (OD), the findings were categorized as follows: positive (OD > 0.170), borderline (OD = 0.130–0.170), and negative (OD < 0.130).

### Ethics

The study protocols and all procedures were reviewed and approved by the Mansoura Institutional Review Board. Written consents were obtained from all patients. The work was done in accordance with the Helsinki Declaration's guidelines.

### Statistical Analyses

Statistical analysis was performed using SPSS software (version 20; SPSS Inc., Chicago, IL, USA). Categorical and continuous parameter differences were expressed using Pearson's chi-square test and Mann–Whitney *U*-test respectively. Our results are expressed as numbers (%) and mean ± SD. Univariate and multivariate logistic regression analyses were performed to identify independent indicators for the presence of various cirrhotic complications. The Bonferroni correction for multiple comparisons was used where appropriate. *P*-values < 0.05 were considered statistically significant.

## Results

**Figure 1:**
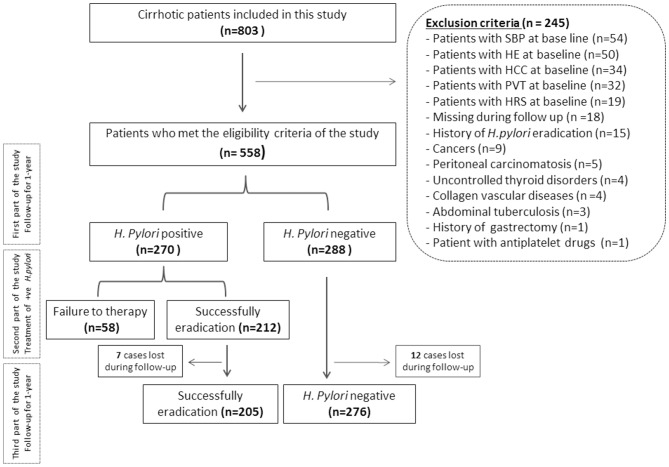
Flowchart of the patients included in this study. SBP, spontaneous bacterial peritonitis; HE, hepatic encephalopathy; HCC, hepatocellular carcinoma; PVT, portal vein thrombosis; HRS, hepatorenal syndrome; *H. pylori, Helicobacter pylori*.

Of the 803 patients with cirrhosis who underwent routine health screening for *H. pylori* presence, 558 patients had liver ultrasound imaging, EGD report, liver function test, and results of *H. pylori* infection and were enrolled in this study. Two-hundred and forty five patients met the exclusion criteria. Of the remaining 558 patients, 288 patients (51.6%) were *H. pylori*-negative and 270 patients (48.4%) were *H. pylori-*positive (Figure 1).

These two groups were compared and were found to have a significant difference in crowding index, serum ammonia, WBCs, CRP, IL-6, TNF-α, NO, and VEGF (all *P* < 0.05), as observed in Table 1.

**Table 1:** Baseline demographic, biochemical, and clinical characteristics of the studied groups.

**Variables**	***H. pylori*-positive *n* = 270**	***H. pylori*-negative *n* = 288**	***P*-value**
Age (years)	53.7 ± 8.8	54.4 ± 7.6	0.31
Sex (M/F)	195/75	209/79	0.98
BMI	25.6 ± 1.9	25.5 ± 1.6	0.5
Current smokers	68 (25.2)	72 (25)	0.96
Regular exercise (%)	10 (4)	12 (4.2)	0.91
Marital status (%)			
Not married^†^	72 (27)	84 (29)	0.6
Married	198 (73)	204 (71)	0.6
Crowding index^‡^	1.19 ± 0.65	1.04 ± 0.53	0.003
Education level (%)			
Low	140 (52)	143 (50)	0.64
Medium	67 (25)	76 (26)	0.79
High	63 (23)	69 (24)	0.78
Employment status (%)			
Not employed	201 (74)	208 (72)	0.6
Employed	69 (26)	80 (28)	0.59
Income (%)			
≥1200 *EGP*	198 (73)	204 (71)	0.6
<1,200 *EGP*	72 (27)	84 (29)	0.59
Diabetes mellitus (%)	65 (24.1)	68 (23.6)	0.89
Hypertension (%)	22 (8.1)	25 (8.6)	0.831
Causes of cirrhosis			
CHC-related liver cirrhosis	268 (99.3)	285 (99)	0.7
CHB- related liver cirrhosis	2 (0. 7)	3 (1)	0.7
Hemoglobin (g/dl)	9.6 ± 0.7	9.7 ± 0.9	0.15
WBCs (× 10^3^/cm^2^)	3.67 ± 1.41	3.14 ± 1.12	<0.001
Platelet count (× 10^3^/cm^2^)	96.4 ± 19.7	94.1 ± 19.1	0.16
ALT (IU/L)	46.4 ± 10	45 ± 10.1	0.1
AST (IU/L)	45.7 ± 12.6	44.3 ± 11.9	0.18
Serum albumin (g/dl)	2.81 ± 0.5	2.79 ± 0.6	0.67
Total Bilirubin (mg/dl)	1.6 ± 0.7	1.5 ± 0.6	0.07
Prothrombin time (sec.)	1.55 ± 0.28	1.53 ± 0.26	0.38
Serum creatinine (mg/dl)	1.11 ± 0.21	1.09 ± 0.18	0.23
eGFR (ml/min/1.73m^2^)	91.4 ± 9.9	89.8 ± 11.1	0.07
Serum ammonia (μmol/l)	118.3 ± 16.05	95.4 ± 7.8	<0.001
CRP (ng/ml)	2.46 ± 0.28	2.18 ± 0.16	<0.001
TNF-α (pg/ml)	10.58 ± 0.67	9.92 ± 0.71	<0.001
IL-6 (pg/ml)	47.17 ± 5.57	42.82 ± 3.2	<0.001
NO (μmol/L)	118.9 ± 7.42	100 ± 8.22	<0.001
VEGF (ng/ml)	0.55 ± 0.084	0.44 ± 0.049	<0.001
AFP (ng/ml)	48.5 ± 11.33	46.8 ± 10.48	0.066
Child–Pugh score	9.75 ± 2.25	8.75 ± 2.12	<0.001
MELD score	20.97 ± 5.05	16.89 ± 3.59	<0.001
Endoscopic findings:			
Esophageal varices [Yes (*n*)]	84 (31)	92 (32)	0.8
Gastric varices [Yes (*n*)]	28 (10)	35 (12)	0.452
PHG [Yes (*n*)]	58 (21)	52 (18)	0.371
GAVE [Yes (*n*)]	19 (7)	17 (6)	0.632
Ascitic fluid analysis			
Positive culture (*n*)	0	0	-
PMN (/mm^3^)	210 ± 24	206 ± 26	0.11
Protein (mg/dl)	397.8 ± 176.8	413 ± 188.5	0.4
Prophylactic use of β-blocker at baseline	56 (21)	63 (22)	0.774
Prophylactic use of *norfloxacin* at baseline	0	0	-
Patients with HCC at baseline	0	0	-
Patients with SBP at baseline	0	0	-
Patients with HE at baseline	0	0	-
Patients with PVT at baseline	0	0	-
Patients with HRS at baseline	0	0	-

Data were expressed as mean ± SD or n (%).

BMI, Basal metabolic index; CHC, chronic hepatitis C; CHB, chronic hepatitis B; EGP, Egyptian Pound; WBCs, white blood cell count; ALT, alanine aminotransferase; AST, aspartate aminotransferase; eGFR, estimated glomerular filltration rate; CRP, C-reactive protein; TNF-α, tumor necrosis factor-α; IL-6; interleukin-6; NO, Nitric oxide; VEGF, vascular endothelial growth factor; AFP, a-fetoprotein; PHG, portal hypertensive gastropathy; GAVE, Gastric antral vascular ectasia; MELD, Model for End-stage Liver Disease; PMN, polymorphonuclear, HCC, hepatocellular carcinoma; SBP, spontaneous bacterial peritonitis; HE, hepatic encephalopathy; PVT, portal vein thrombosis, HRS, hepatorenal syndrome.

† Not married, involves widowed, divorced, separated, or single.

‡ *Crowding index, number of individuals/room excluding the kitchen and bathroom (33)*.

### Results of *H. pylori*-Positive Patients 1-Year Follow-Up

There was a significant increase in serum ammonia, CRP, IL-6, TNF-α, NO, VEGF, AFP, ascitic PMNs, and Child–Pugh score as well as there was a significant decrease in WBCs, platelet count, AST, serum albumin, eGFR, and ascitic fluid protein (all *P* < 0.05) as highlighted in Table 2.

**Table 2:** Demographic, biochemical, and clinical characteristics of the studied groups after a 1-year follow-up.

**Variables**	***H. pylori*-positive *n* = 270**	***H. pylori*-negative *n* = 288**	***P*-value**
	**Follow-up at** **1 year**	**Follow-up at** **1 year**	
Age (years)	54.7 ± 8.8	55.4 ± 7.6	P1 = 0.187 P2 = 0.115 P3 = 0.31
Sex (M/F)	195/75	209/79	P3 = 0.973
BMI	25.7 ± 1.6	25.6 ± 1.4	P1 = 0.509 P2 = 0.425 P3 = 0.432
Hemoglobin (g/dl)	9.65 ± 0.6	9.68 ± 0.7	P1 = 0.373 P2 = 0.766 P3 = 0.588
WBCs (× 10^3^/cm^2^)	3.32 ± 1.08	2.97 ± 0.78	P1 = 0.001 P2 = 0.035 P3 < 0.001
Platelet count (× 10 ^3^/cm^2^)	89.8 ± 16	85 ± 13.8	P1 < 0.001 P2 < 0.001 P3 < 0.001
ALT (IU/L)	44.9 ± 12.1	39.7 ± 8.5	P1 = 0.117 P2 < 0.001 P3 < 0.001
AST (IU/L)	42.4 ± 10.1	40 ± 8.8	P1 < 0.001 P2 < 0.001 P3 = 0.003
Serum albumin (g/dl)	2.72 ± 0.5	2.62 ± 0.6	P1 = 0.037 P2 < 0.001 P3 = 0.034
Total Bilirubin (mg/dl)	1.7 ± 0.5	1.6 ± 0.6	P1 = 0.057 P2 = 0.046 P3 = 0.034
Prothrombin time (sec.)	1.59 ± 0.24	1.34 ± 0.29	P1 = 0.075 P2 < 0.001 P3 < 0.001
Serum creatinine (mg/dl)	1.12 ± 0.12	1.02 ± 0.19	P1 = 0.497 P2 < 0.001 P3 < 0.001
eGFR (ml/min/1.73m^2^)	89.2 ± 13.1	82.4 ± 11.6	P1 = 0.028 P2 < 0.001 P3 < 0.001
Serum ammonia (μmol/l)	130.9 ± 13.04	99.3 ± 8.4	P1 < 0.001 P2 < 0.001 P3 < 0.001
CRP (ng/ml)	2.82 ± 0.22	2.23 ± 0.19	P1 < 0.001 P2 < 0.001 P3 < 0.001
TNF-α (pg/ml)	11.26 ± 0.54	9.89 ± 0.66	P1 < 0.001 P2 = 0.6 P3 < 0.001
IL-6 (pg/ml)	53.4 ± 4	43 ± 2.3	P1 < 0.001 P2 = 0.439 P3 < 0.001
NO (μmol/L)	127.9 ± 5.66	103 ± 6.22	P1 < 0.001 P2 < 0.001 P3 < 0.001
VEGF (ng/ml)	0.67 ± 0.09	0.46 ± 0.05	P1 < 0.001 P2 < 0.001 P3 < 0.001
AFP (ng/ml)	75.4 ± 30.52	68 ± 28.32	P1 < 0.001 P2 < 0.001 P3 < 0.001
Child–Pugh score	10.25 ± 2.75	9 ± 2.5	P1 = 0.021 P2 = 0.196 P3 < 0.001
MELD score	21.62 ± 4.87	16.9 ± 2.6	P1 = 0.129 P2 = 0.97 P3 < 0.001
Endoscopic findings:			
Patients develop new EV	29/186 (15.6)	16/196 (8.1)	P3 = 0.023
Patients develop new GV	18/242 (7.4)	7/253 (2.8)	P3 = 0.02
Patients develop new PHG	15/212 (7.1)	6/236 (2.5)	P3 = 0.022
Patients develop new GAVE	11/251 (4.4)	4/271 (1.5)	P3 = 0.049
Ascitic fluid analysis			
Positive culture (n)	14 (6.1)	4 (1.9)	P3 = 0.027
PMN (/mm^3^)	280 ± 85	245 ± 55	P1 < 0.001 P2 < 0.001 P3 < 0.001
Protein (mg/dl)	368.5 ± 155	403 ± 164	P1 = 0.064 P2 = 0.549 P3 = 0.017
Prophylactic use of β-blocker	61 (23)	69 (24)	P1 = 0.575 P2 = 0.569 P3 = 0.781
Prophylactic use of *norfloxacin*	8 (3)	7 (2)	P3 = 0.449
Patients develop HCC	16 (5.9)	7 (2.4)	P3 = 0.037
Patients develop SBP	33/230 (14)	14/209 (6.7)	P3 = 0.006
Patients develop SBP recurrence	15/33 (45.5)	2/14 (14.3)	P3 = 0.044
Patients develop HE	75 (27.8)	42 (14.6)	P3 < 0.001
Patients develop PVT	31 (11.5)	15 (5)	P3 = 0.005
Patients develop HRS	30 (11)	8 (3)	P3 < 0.001

Data were expressed as mean ± SD or n (%).

P1: H. pylori-positive at baseline vs. at 1 year follow-up.

P2: H. pylori-negative at baseline vs. at 1 year follow-up.

P3: H. pylori-positive vs. H. pylori-negative at 1 year follow-up.

*BMI, Basal metabolic index; WBCs, white blood cell count; ALT, alanine aminotransferase; AST, aspartate aminotransferase; eGFR, estimated glomerular filtration rate; CRP, C-reactive protein; TNF-α, tumor necrosis factor-α; IL-6, interleukin-6; NO, Nitric oxide; VEGF, vascular endothelial growth factor; AFP, α-fetoprotein; EV, esophageal varices; GV, gastric varices; PHG, portal hypertensive gastropathy; GAVE, Gastric antral vascular ectasia; MELD, Model for End-stage Liver Disease; PMN, polymorphonuclear; HCC, hepatocellular carcinoma; SBP, spontaneous bacterial peritonitis; HE, hepatic encephalopathy; PVT, portal vein thrombosis; HRS, hepatorenal syndrome*.

### Results of *H. pylori*-Negative Patients 1-Year Follow-Up

There was a significant increase in total bilirubin, serum ammonia, CRP, VEGF, NO, AFP, and ascitic PMNs as well as there was a significant decrease in WBCs, platelet count, ALT, AST, serum albumin, prothrombin time, serum creatinine, and e GFR (all *P* < 0.05) as listed in Table 2.

### Comparison of 1-Year Follow-Up Results in Both Groups

There was a significant increase WBCs, platelet count, ALT, AST, serum albumin, prothrombin time, serum creatinine, eGFR, serum ammonia, CRP, IL-6, TNF-α, NO, AFP, VEGF levels, Child–Pugh score, and ascitic PMNs as well as there was a significant decrease in ascitic fluid protein in *H. pylori*-positive vs. negative patients (all *P* < 0.05), as observed in Table 2.

There was a significant increase in the incidence of esophageal varices (EV), gastric varices (GV), PHG, GAVE, HCC, SBP and its recurrence, HE, PVT, and HRS in *H. pylori*-positive vs. negative patients (all *P* < 0.05), as highlighted in Table 2.

According to ascitic fluid analysis, among the 47 patients who developed SBP in both groups, 18 (38%) had positive ascitic fluid cultures; the isolated organism was only Escherichia coli (*E. coli*). The rest of the SBP patients had culture-negative SBP (*n* = 29). Ascitic fluid with positive cultures was significantly increased in *H. pylori-positive vs. negative* patients (*P* = 0.027) as demonstrated in Table 2.

### 3-Months Follow-Up Results After *H. pylori*-Positive Patients Treatment

There was a significant decrease in WBCs count, serum albumin, creatinine, ammonia, CRP, IL-6, TNF-α, NO, AFP, and VEGF levels as well as there was a significant increase in hemoglobin level (all *P* < 0.001), as reported in Table 3.

**Table 3:** Changes in biochemical, clinical, and demographic characteristics of the *H. pylori* positive group after 3 months of therapy.

**Variables**	**Baseline (*n* = 212)**	**After 3 months of therapy**	***P*-value**
Age (years)	53 ± 7.7	-	-
Sex (M/F)	154/58	-	-
BMI	25.7 ± 1.72	25.64 ± 1.39	0.693
Hemoglobin (g/dl)	9.5 ± 0.6	9.9 ± 0.5	<0.001
WBCs (× 10^3^/cm^2^)	3.51 ± 0.99	3.16 ± 0.61	<0.001
Platelet count (× 10^3^/cm^2^)	93.6 ± 11.9	94.5 ± 14.8	0.491
ALT (IU/L)	45.6 ± 12.8	47.5 ± 8.8	0.076
AST (IU/L)	44.4 ± 8.8	43 ± 10	0.127
Serum albumin (g/dl)	2.78 ± 0.54	2.75 ± 0.52	<0.001
Total Bilirubin (mg/dl)	1.7 ± 0.5	1.68 ± 0.7	0.735
Prothrombin time (sec.)	1.58 ± 0.26	1.54 ± 0.18	0.066
Serum creatinine (mg/dl)	1.13 ± 0.13	1.08 ± 0.17	<0.001
eGFR (ml/min/1.73 m^2^)	87.5 ± 12.7	85.5 ± 12.2	0.1
Serum ammonia (μmol/l)	131.5 ± 13.52	93.61 ± 10.6	<0.001
CRP (ng/ml)	2.79 ± 0.22	2.04 ± 0.22	<0.001
TNF-α (pg/ml)	11.32 ± 0.49	9.27 ± 0.83	<0.001
IL-6 (pg/ml)	53.29 ± 4.03	44.66 ± 3.72	<0.001
NO (μmol/L)	128 ± 5.35	101.67 ± 9.88	<0.001
VEGF (ng/ml)	0.66 ± 0.089	0.48 ± 0.05	<0.001
AFP (ng/ml)	65.3 ± 23.41	58.2 ± 22.17	<0.001
Child–Pugh score	9.5 ± 2.2	9.3 ± 2	0.327
MELD score	21.35 ± 4.96	20.45 ± 5.01	0.064

Data were expressed as mean ± SD or n (%).

*BMI, Basal metabolic index; WBCs, white blood cell count; ALT, alanine aminotransferase; AST, aspartate aminotransferase; eGFR, estimated glomerular filtration rate; CRP, C-reactive protein; TNF-α, tumor necrosis factor-α; IL-6, interleukin-6; NO, Nitric oxide; VEGF, vascular endothelial growth factor; AFP, α-fetoprotein; MELD, Model for End-stage Liver Disease*.

### Data From an Extra 1-Year Follow-Up Assessment After *H. pylori* Eradication

There was no significant changes regarding all variables as well as there was no statistically significant difference in the incidence of EV, GV, PHG, GAVE, HCC, SBP and their recurrence, HE, PVT, and HRS in *H. pylori*-positive patients with eradication vs. *H. pylori*-negative patients (all *P* > 0.05), as observed in Table 4.

**Table 4:** Baseline demographic, biochemical, and clinical characteristics of the studied groups for 1 year after treatment of *H. pylori-*positive patients (Successful *H. pylori* treatment).

**Variables**	**Successful** ***H. pylori*** **treatment** ***(n*** **=** **205)**		***H. pylori*****-negative (*****n*** **=** **276)**		***P*-value**
	**Baseline**	**Follow-up at** **1-year**	**Baseline**	**Follow-up at 1-year**	
Age (years)	54.7 ± 8.8	55.7 ± 8.8	55.4 ± 7.6	56.4 ± 7.6	P1 = 0.253 P2 = 0.123 P3 = 0.251 P4 = 0.351
Sex (M/F)	147/58	-	195/81	-	P3 = 0.933
BMI	25.53 ± 1.36	25.58 ± 1.39	25.62 ± 1.45	25.7 ± 1.5	P1 = 0.713 P2 = 0.524 P3 = 0.49 P4 = 0.371
Hemoglobin (g/dl)	9.8 ± 0.45	9.7 ± 0.5	9.6 ± 0.65	9.8 ± 0.7	P1 = 0.034 P2 < 0.001 P3 < 0.001 P4 = 0.082
WBCs (× 10^3^/cm^2^)	3.12 ± 0.55	3.35 ± 0.58	2.85 ± 0.63	3.31 ± 0.71	P1 < 0.001 P2 < 0.001 P3 < 0.001 P4 = 0.51
Platelet count (× 10^3^/cm^2^)	90.5 ± 14	93.4 ± 16	89 ± 13.9	92 ± 15	P1 = 0.052 P2 = 0.015 P3 = 0.244 P4 = 0.326
ALT (IU/L)	45.3 ± 8.2	43.5 ± 10	40.2 ± 8.7	42 ± 9.1	P1 = 0.047 P2 = 0.018 P3 < 0.001 P4 = 0.087
AST (IU/L)	42 ± 9.5	40.4 ± 10.2	39 ± 8.5	41.3 ± 10.4	P1 = 0.101 P2 = 0.005 P3 < 0.001 P4 = 0.345
Serum albumin (g/dl)	2.62 ± 0.52	2.56 ± 0.53	2.58 ± 0.55	2.6 ± 0.62	P1 = 0.248 P2 = 0.689 P3 = 0.42 P4 = 0.457
Total Bilirubin (mg/dl)	1.61 ± 0.7	1.65 ± 0.65	1.68 ± 0.62	1.7 ± 0.65	P1 = 0.549 P2 = 0.712 P3 = 0.247 P4 = 0.405
Prothrombin time	1.51 ± 0.17	1.55 ± 0.2	1.3 ± 0.29	1.51 ± 0.3	P1 = 0.03 P2 < 0.001 P3 < 0.001 P4 = 0.1
Serum creatinine (mg/dl)	1.05 ± 0.16	1.1 ± 0.21	1.01 ± 0.19	1.09 ± 0.2	P1 = 0.007 P2 < 0.001 P3 = 0.015 P4 = 0.596
eGFR (ml/min/1.73 m^2^)	84.2 ± 10.8	87 ± 12.5	81.2 ± 11.2	86 ± 12	P1 = 0.016 P2 < 0.001 P3 = 0.003 P4 = 0.375
Serum ammonia (μmol/l)	91.72 ± 9.5	93 ± 9.3	93.3 ± 8.2	94.1 ± 8.7	P1 = 0.169 P2 = 0.267 P3 = 0.052 P4 = 0.184
CRP (ng/ml)	2 ± 0.21	2.11 ± 0.25	2.12 ± 0.16	2.14 ± 0.18	P1 < 0.001 P2 = 0.168 P3 < 0.001 P4 = 0.127
TNF-α (pg/ml)	9.23 ± 0.81	9.3 ± 0.83	9.35 ± 0.65	9.41 ± 0.68	P1 = 0.388 P2 = 0.29 P3 = 0.072 P4 = 0.111
IL-6 (pg/ml)	42.53 ± 3.51	43.2 ± 3.7	42.8 ± 2.4	43.7 ± 3.8	P1 = 0.061 P2 < 0.001 P3 = 0.317 P4 = 0.15
NO (μmol/L)	100.53 ± 9.75	103 ± 9.8	101 ± 6.11	104 ± 6.4	P1 = 0.011 P2 < 0.001 P3 = 0.517 P4 = 0.053
VEGF (ng/ml)	0.45 ± 0.05	0.46 ± 0.06	0.45 ± 0.06	0.47 ± 0.06	P1 = 0.068 P2 < 0.001 P3 = 0.99 P4 = 0.071
AFP (ng/ml)	54.6 ± 19.55	59.4 ± 20.54	52 ± 19.85	58 ± 21.42	P1 = 0.016 P2 < 0.001 P3 = 0.07 P4 = 0.471
Child–Pugh score	9.2 ± 2.1	9.4 ± 2.3	9.1 ± 2.2	9.3 ± 2.4	P1 = 0.358 P2 = 0.308 P3 = 0.616 P4 = 0.646
MELD score	20.31 ± 5	21.24 ± 5.1	16.5 ± 2.5	20.8 ± 3.1	P1 = 0.063 P2 < 0.001 P3 < 0.001 P4 = 0.242
Endoscopic findings:					
Patients develop new OV	-	12/136 (8.8)	-	14/171 (8.2)	P4 = 0.851
Patients develop new GV	-	5/175 (2.9)	-	7/246 (2.8)	P4 = 0.952
Patients develop new PHG	-	5/145 (3.4)	-	8/225 (3.5)	P4 = 0.959
Patients develop new GAVE	-	3/186 (1.6)	-	4/257 (1.55)	P4 = 0.967
Ascitic fluid analysis					
Positive culture (n)	-	5 (2.7)	-	6 (2.3)	P4 = 0.788
PMN (/mm^3^)	214 ± 28	274 ± 45	212 ± 30	281 ± 55	P1 < 0.001 P2 < 0.001 P3 = 0.457 P4 = 0.137
Protein (mg/dl)	362 ± 158	351 ± 144	392 ± 178	365 ± 159	P1 = 0.463 P2 = 0.061 P3 = 0.057 P4 = 0.321
Prophylactic use of β-blocker	57 (28)	53 (26)	61 (22)	64 (23)	P1 = 0.649 P2 = 0.779 P3 = 0.131 P4 = 0.448
Prophylactic use of *norfloxacin*	5 (2.4)	4 (1.9)	6 (2.2)	5 (1.8)	P1 = 0.727 P2 = 0.737 P3 = 0.885 P4 = 0.936
Patients develop new HCC	-	7/194 (3.6)	-	9/270 (3.3)	P4 = 0.861
Patients develop new SBP	-	14/185 (7.6)	-	19/263 (7.2)	P4 = 0.873
Patients develop SBP recurrence	-	2/14 (14.3)	-	3/19 (15.8)	P4 = 0.91
Patients develop new HE	-	20/145 (13.8)	-	32/238 (13.4)	P4 = 0.912
Patients develop new PVT	-	13/180 (7.2)	-	14/263 (5.3)	P4 = 0.411
Patients develop new HRS	-	8/181 (4.4)	-	10/268 (3.7)	P4 = 0.71

Data were expressed as mean ± SD or n (%).

P1: Successful H. pylori treatment at baseline vs. at 1 year follow-up.

P2: H. pylori-negative at baseline vs. at 1 year follow-up.

P3: Successful H. pylori treatment vs. H. pylori-negative at baseline.

P4: Successful H. pylori treatment vs. H. pylori-negative at 1 year follow-up.

*BMI, Basal metabolic index; WBCs, white blood cell count; ALT, alanine aminotransferase; AST, aspartate aminotransferase; eGFR, estimated glomerular filtration rate; CRP, C-reactive protein; TNF-α, tumor necrosis factor-α; IL-6, interleukin-6; NO, Nitric oxide; VEGF, vascular endothelial growth factor; AFP, α-fetoprotein; EV, esophageal varices; GV, gastric varices; PHG, portal hypertensive gastropathy; GAVE, Gastric antral vascular ectasia; MELD, Model for End-stage Liver Disease; PMN, polymorphonuclear; HCC, hepatocellular carcinoma; SBP, spontaneous bacterial peritonitis; HE, hepatic encephalopathy; PVT, portal vein thrombosis; HRS, hepatorenal syndrome*.

According to ascitic fluid analysis, among the 33 patients who develop SBP in both groups, 11 (33%) had positive ascitic fluid cultures; the isolated organism was only Escherichia coli (*E. coli*). The rest of the SBP patients had culture-negative SBP (*n* = 22). No significant differences were found between the two groups (*P* = 0.873) as shown in Table 4.

All patients with PVT in both groups had been subjected to measurements of activated partial thromboplastin time, fibrinogen, protein C, protein S, antithrombin III, serum homocysteine, D-dimer, antinuclear antibody, anticardiolipin IgG antibodies, anti-double-strand DNA after 1 year of the follow-up and after 1 year of *H. pylori* eradication. No significant difference between the two groups was evident in both conditions. The details of diagnostic biochemical criteria were described in Tables S1, S2.

### Univariate and Multivariate Regression Analysis Models Predicting Different Cirrhotic Complications Within the Follow-Up Period

Univariate analysis revealed that the presence of *H. pylori* infection was associated independently with the development of SBP, PVT, and HCC (all *P* < 0.05) while multivariate regression analysis model reported that the presence of *H. pylori* infection was associated independently with the development of only PVT and HCC, after adjustment for age, BMI, inflammatory markers (CRP, IL-6, and TNF-α), vascular mediators (VEGF and NO), Child-Pugh score and MELD score as shown in Table 5.

**Table 5:** Univariate and multivariate regression analysis models predict cirrhotic complications within the follow-up period after adjusting for multiple confounding variables.

	**Parameters**	**Univariate regression analysis**		**Multivariate regression analysis**	
		**OR (95% CI)**	***P*-value**	**OR (95% CI)**	***P*-value**
HCC	*H. pylori*-positive	2.53 (1.02–6.25)	0.044	0.11 (0.01–0.88)	0.037
	NO	1.07 (1.02–1.11)	0.002	1.15 (1.04–1.26)	0.004
	VEGF	18.36 (1.94–173.65)	0.024	-	-
PVT	*H. pylori*-positive	1.03 (1.01–1.05)	<0.001	1.72 (1.18–4.53)	0.043
	Amonia	2.98 (1.6–5.56)	<0.001	1.04 (1.02–1.06)	<0.001
SBP	*H. pylori*-positive	2.73 (1.4–5.21)	0.003	-	-
	IL-6	1.1 (1.04–1.17)	0.001	-	-
	TNF	3.53 (2.12–5.85)	<0.001	2.81 (1.5–5.18)	<0.001
	CRP	12.02 (3.75–38.47)	<0.001	-	-

Data were expressed as median (range).

*CRP, C-reactive protein; TNF-α, tumor necrosis factor-α; IL-6, interleukin-6; NO, Nitric oxide; VEGF, vascular endothelial growth factor; HCC, hepatocellular carcinoma; SBP, spontaneous bacterial peritonitis; PVT, portal vein thrombosis*.

### Incidence Rate (IR), Incidence Rate Difference (IRD), and Incidence Rate Ratio (IRR) of Cirrhotic Complications Resulting From Multivariate Regression Analysis Models Before and After Therapy Were Demonstrated as Follows

As regards to PVT, the IR was 0.115 (95% CI, 0.078–0.163) before therapy, and changed to 0.072 (95% CI, 0.0385–0.124) after therapy. The IRD was 0.043 (95% CI, −0.016–0.102) with *P* = 0.042. The IRR was 1.59 (95% CI, 0.81–3.31).

As regards to HCC, the IR was 0.059 (95% CI, 0.034–0.096) before therapy, and changed to 0.036 (95% CI, 0.015–0.074) after therapy. The IRD was 0.023 (95% CI, −0.018–0.064) with *P* = 0.037. The IRR was 1.64 (95% CI, 0.64–4.72).

## Discussion

*H. pylori* infection leads to an increase of proinflammatory cytokines interleukin (IL)-1, IL-2, IL-4, IL-6, IL-8, IL-10, IL-17, TNF-α, and interferon-β (36). It has been hypothesized that infection with *H. pylori* has a systemic effect via the activity of these cytokines and exacerbation of different inflammatory reactions (7).

Portal hypertension (PH) plays a major role in the development of cirrhotic complications. In this regard, recent papers have suggested that *H. pylori* could lead to a condition of chronic vasculitis, thus causing endothelial dysfunction (37). The involvement of PV may prompt the increased stiffness of the vessel, with a decreased capacity to adapt to blood flow changes, leading to increased portal pressure, regardless of inadequate direct experimental evidence (38, 39). The systemic inflammatory conditions, actuated by *H. pylori*, enhance NO production. Recent research has reported that the concentration of NO was increased in cirrhotic *H. pylori*-positive patients (40). This finding was concurrent with our outcomes. NO is a common vasodilator and is implicated in the regulation of systemic and splanchnic hemodynamics in PH (41, 42). In addition, lipopolysaccharide of *H. pylori*, in a cultured cell model of liver tissue, could initiate a microvascular inflammatory response by stimulating the inducible nitric oxide synthase (iNOS), a mechanism that may share in the pathogenesis of PH (43).

*H. pylori* is believed to magnify the expression of VEGF, via a signaling pathway involving the MEK-ERK and NF-kB cascade (44, 45). Elevated VEGF concentration was related to a significant increment in neo-angiogenesis as estimated by of CD34-positive micro-vessels determination. Increased levels of the VEGF in this study corroborate these earlier findings (44). A strong correlation between serum VEGF levels and PH was demonstrated, and the proof that *H. pylori* may lead to a local increment in VEGF in gastric tissue might bolster a unifying hypothesis in which experimental and human models help a direct mediation in VEGF-induced PH (46, 47).

The impact of *H. pylori* on PH may be multifactorial. Alterations in the vasodilatation dynamics, endothelial dysfunction, and vascular overgrowth are the most engaging speculations about these issues.

The results of this study indicate that the incidence of PVT increased in *H. pylori*-positive patients. The possible explanation may be due to the fact that *H. Pylori* secretes a neutrophilic activating protein that leads to neutrophilic infiltration of vascular walls resulting in venous thrombosis (48). The bacterium was reported to enhance the secretion of adhesion molecules, for example, E-selectin, intercellular adhesion molecule-1 **(**ICAM-1), and vascular cell adhesion molecule-1 (VCAM-1), as well as growth-related oncogene alpha, IL-6, and IL-8 (49). All these agents can enhance the induction of neutrophils through the endothelium, an occasion which is an initiating etiology of endothelial dysfunction. Moreover, increased stiffness of the PV induced by *H. pylori* (39), the “angiogenetic drive” of H. pylori (50) and the reduction in mean portal flow velocity in cirrhotics (51) support our hypothesis in the development of PVT.

The most important clinically relevant finding was the increased incidence of HCC in *H. pylori*-positive patients. These relationships may partly be explained by the role of *H. pylori* infection in activating the transforming growth factor β1-dependent oncogenic pathway, affecting the equilibrium between hepatocyte proliferation and apoptosis in experimental models (52). These results were reported by Zhang et al. that *H. pylori* infection leads to a direct cytopathic effect on HepG2 hepatoma cells by upregulating the expression of various proteins associated with signal transduction and gene transcription (53). Excess generation of NO is well-recognized as an essential step initiating neoplastic transformation (54). In addition, NO plays a pivotal role in the development of HCC and its progression (55). In this study, the multivariate analysis revealed that NO can be considered as an independent risk factor for the development of HCC in *H. pylori*-positive patients. The role of *H. pylori* in production of NO was previously discussed (40, 43).

In addition, VEGF is a master regulator of angiogenesis in malignant and normal tissues. It plays an essential function in enhancing the proliferation of endothelial cells, thus favoring neovascularization within and around malignant cells. It participates in many other conditions such as activation of receptors related to the proliferation of tumor cells and recruitment of endothelial cells (56, 57). *H. pylori* is associated with the production of VEGF (44), which may lead to HCC development. No doubt these findings will be much scrutinized, but there are some immediate dependable conclusions for the role of *H. pylori* infection and the development of HCC in this study.

Our findings reported a significant decrease in serum ammonia, pro-inflammatory mediators, NO, and VEGF levels as well as decreased incidence of all studied cirrhotic complications after *H. pylori* infection treatment. Overall, this study strengthens the idea that *H. pylori* infection induces hepatic decompensation. The precise mechanism of *H. pylori* in hepatic decompensation remains to be elucidated. This new understanding should help in improving the predictions of *H. pylori* infection impact on liver cirrhosis.

If we assume the treatment with different antibiotics/PPIs is the cause of the decrease in the inflammatory parameters, they would have increased again after the stoppage of the treatment. Even after the 1 year follow up these mediators/markers didn't show any increase again in comparison with the *H. pylori* negative patients. This means that *H. pylori* was the only factor behind this elevation.

This study, to the best of our knowledge, is the first to contribute to this growing area of research by exploring the impact of *H. pylori* on liver cirrhosis. These findings have significant implications in the understanding of how *H. pylori* was implicated in the pathogenesis of various cirrhotic complications.

The generalizability of these results are subject to certain limitations. First; single-center study. Second; GI endoscopy was used to diagnose PHG and GAVE with no histopathological assessment. Third; after therapy and successful *H. pylori* eradication, recurrence of SBP may have been affected by the use of PPI (58) and the prophylactic use of β-blockers (58, 59). Fourth; some patients were non-adherent to the standard recommendations (at least to receive norfloxacin for secondary prophylaxis of SBP and prophylactic use of β-blockers for varices). Fifth, several studies have obtained controversial results, on the relationship between *H. pylori* infection and cirrhosis, on the basis of etiology (60). Because most of the cases have the same etiology which is hepatitis C virus, we cannot incorporate the different etiologies in our multivariate analysis, or correlate them with our results. Finally, the probability of false negatives becoming positives during reassessment/follow-up may occur, but repeated and strict measures were followed to avoid this bias.

In spite of its limitations, the study certainly adds to our understanding of the role of *H. pylori* in different complications of liver cirrhosis. Even if there were non- *H. pylori* factors contributing to the outcome seen, we think it is a great achievement to draw the attention of the clinicians to the important role played by *H. pylori* in the pathophysiology of cirrhotic complications and consequently the importance of *H. pylori* treatment and eradication in these patients.

In conclusion, *H. pylori* infection was evidently related to increased incidence of various cirrhotic complications, especially hepatocellular carcinoma and portal vein thrombosis development through increased secretion of a lot of inflammatory markers and vascular mediators. Moreover, its eradication may reduce the incidence of these complications.

## Data Availability Statement

The data that support the findings of this study have restrictions and so are not publicly available. Data are however available from the authors upon reasonable request.

## Ethics Statement

The studies involving human participants were reviewed and approved by Ethics Committee of the Mansoura University, Egypt (Approval no. R.19.08.573). The patients/participants provided their written informed consent to participate in this study.

## Author Contributions

Guarantor of the article: AA-R. AA-R and NM acquired, analyzed, and interpreted data, performed statistical analysis, and wrote, edited, and reviewed the manuscript. MA and AS recruited and followed up with patients, acquired, analyzed, and interpreted data, performed statistical analysis, and critically revised the manuscript. AH and AT recruited and followed up with patients, acquired, analyzed, and interpreted data, critically revised the manuscript. RElh and RElz acquired, analyzed and interpreted data, underwent laboratory investigations, and revised the manuscript. WE and NE-W acquired, analyzed, and interpreted data, performed statistical analysis, underwent laboratory investigations, and critically revised the manuscript. All authors approved the final version of the article, including the authorship list.

### Conflict of Interest

The authors declare that the research was conducted in the absence of any commercial or financial relationships that could be construed as a potential conflict of interest.

## Abbreviations

BMI: Basal metabolic index
CHC: chronic hepatitis C
CHB: chronic hepatitis B
WBCs: white blood cell count
ALT: alanine aminotransferase
AST: aspartate aminotransferase
eGFR: estimated glomerular filtration rate
CRP: C-reactive protein
TNF-α: tumor necrosis factor-α
IL-6: interleukin-6
NO: Nitric oxide
VEGF: vascular endothelial growth factor
AFP: α-fetoprotein
PPIs: proton-pump inhibitors
PHG: portal hypertensive gastropathy
GAVE: Gastric antral vascular ectasia
MELD: Model for End-stage Liver Disease
PMN: polymorphonuclear
HCC: hepatocellular carcinoma
SBP: spontaneous bacterial peritonitis
HE: hepatic encephalopathy
PVT: portal vein thrombosis
HRS: hepatorenal syndrome.
