# Supplementary material for: Helicobacter pylori as an Initiating Factor of Complications in Patients With Cirrhosis: A Single-Center Observational Study
Source: Front Med (Lausanne). 2020 Mar 24;7:96. doi: 10.3389/fmed.2020.00096 (PMC7105722; doi:10.3389/fmed.2020.00096)
Supplement: Supplementary file 1 [file Data_Sheet_1.PDF]

Supplementary data

Table S1. Biochemical characteristics of *H. pylori*-positive patients with portal vein thrombosis development throughout the study.

Parameters	Baseline	Patients with PVT 1-year follow up before therapy (*n*=31)	Patients with PVT followed up for one further year after therapy (*n*=13)	*P* value
Protein S (IU/dL)	84.7±7.3	85.8±7.6	86.6±7.3	P1=NS<br>P2=NS<br>P3=NS
Protein C (IU/dL)	81.3±7.8	82.4±7.9	84.2±7.9	P1=NS<br>P2=NS<br>P3=NS
Antithrombin III	82.2±5.3	82.5±5.4	84.3±5.5	P1=NS<br>P2=NS<br>P3=NS
Fibrinogen levels (mg/dl)	180±57	182±59	183±57	P1=NS<br>P2=NS<br>P3=NS
Factor VIII (ng/ml)	100.5±9.1	101.8±9.2	103.9±9.4	P1=NS<br>P2=NS<br>P3=NS
Homocysteine (μmol/L)	13±0.65	13.1±0.6	13.2±0.7	P1=NS<br>P2=NS<br>P3=NS
PAI-1 (ng/ml)	10.6±2.4	10.7±2.5	10.5±2.55	P1=NS<br>P2=NS<br>P3=NS

D-dimer (ng/ml)	438±291	445±290	441±288	P1=NS<br>P2=NS<br>P3=NS
Portal flow velocity (cm/s)	12.6±3.5	12.8±3.6	12.9±3.6	P1=NS<br>P2=NS<br>P3=NS
Anti-dsDNA positive (*n*) (positive >20 IU/ml)	0	0	0	-
ANA positive (*n*) (positive ≥1.2)	0	0	0	-
ACA-IgG positive (*n*) (positive ≥10 U/ml)	0	0	0	-

Data were expressed as mean±SD

PAI, plasminogen activator inhibitor; ANA, antinuclear antibody; Anti-dsDNA, anti-double-strand DNA; ACA-IgG, anticardiolipin IgG antibody; NS, not significant.

P1 H. pylori-positive at baseline vs. at one year follow-up before therapy.

P2 H. pylori-positive at baseline vs. at one further year after therapy

P3 H. pylori-positive at one year follow-up before therapy vs. at one further year after therapy

Table S2. Biochemical characteristics of *H. pylori*-negative patients with portal vein thrombosis development throughout the study.

Parameters	Baseline	Patients with PVT 1-year follow up (*n*=15)	Patients with PVT followed up for one further year (*n*=14)	*P* value
Protein S (IU/dL)	82.9±7.2	83.8±7.1	86.8±7.4	P1=NS<br>P2=NS<br>P3=NS
Protein C (IU/dL)	78.4±7.5	79.5±7.6	83.8±7.8	P1=NS<br>P2=NS<br>P3=NS
Antithrombin III	75.6±4.7	76.2±4.9	81.6±5.2	P1=NS<br>P2=NS<br>P3=NS
Fibrinogen levels (mg/dl)	170±49	177±51	181±58	P1=NS<br>P2=NS<br>P3=NS
Factor VIII (ng/ml)	95.8±8.3	96.5±8.5	100.7±9.1	P1=NS<br>P2=NS<br>P3=NS
Homocysteine (μmol/L)	12.1±0.4	12.5±0.5	12.9±0.7	P1=NS<br>P2=NS<br>P3=NS
PAI-1 (ng/ml)	10.1±2	10.3±2.1	10.4±2.4	P1=NS<br>P2=NS<br>P3=NS
D-dimer (ng/ml)	414±255	418±261	429±275	P1=NS<br>P2=NS

				P3=NS
Portal flow velocity (cm/s)	12.8±3.7	12.9±3.8	13±3.8	P1=NS<br>P2=NS<br>P3=NS
Anti-dsDNA positive (*n*) (positive >20 IU/ml)	0	0	0	-
ANA positive (*n*) (positive ≥1.2)	0	0	0	-
ACA-IgG positive (*n*) (positive ≥10 U/ml)	0	0	0	-

Data were expressed as mean±SD

PAI, plasminogen activator inhibitor; ANA, antinuclear antibody; Anti-dsDNA, anti-double-strand DNA; ACA-IgG, anticardiolipin IgG antibody; NS, not significant.

P1 *H. pylori*-negative at baseline vs. at one year follow-up.

P2 *H. pylori*-negative at baseline vs. at one further year.

P3 *H. pylori*-negative at one year follow-up vs. at one further year.
